# Attribution of undesirable character traits, rather than trait-based dehumanization, predicts punishment decisions

**DOI:** 10.1098/rsos.240087

**Published:** 2024-07-17

**Authors:** Robert A. Brennan, Florence E. Enock, Harriet Over

**Affiliations:** ^1^ Department of Psychology, University of York, York YO10 5DD, UK; ^2^ The Alan Turing Institute, British Library, 96 Euston Road, London NW1 2DB, UK

**Keywords:** dehumanization, intergroup bias, intergroup harm, punishment, social cognition

## Abstract

Previous work has reported that the extent to which participants dehumanized criminals by denying them uniquely human character traits such as refinement, rationality and morality predicted the severity of the punishment endorsed for them. We revisit this influential finding across six highly powered and pre-registered studies. First, we conceptually replicate the effect reported in previous work, demonstrating that our method is sensitive to detecting relationships between trait-based dehumanization and punishment should they occur. We then investigate whether the apparent relationship between trait-based dehumanization and punishment is driven by the desirability of the traits incorporated into the stimulus set, their perceived humanness, or both. To do this, we asked participants to rate the extent to which criminals possessed uniquely human traits that were either socially desirable (e.g. cultured and civilized) or socially undesirable (e.g. arrogant and bitter). Correlational and experimental evidence converge on the conclusion that apparent evidence for the relationship between trait-based dehumanization and punishment is better explained by the extent to which participants attribute socially desirable attributes to criminals rather than the extent to which they attribute uniquely human attributes. These studies cast doubt on the hypothesized causal relationship between trait-based dehumanization and harm, at least in this context.

## 1. Introduction

Understanding the motivations that lead to intergroup harm has been a driving force behind social psychology since its conception [1,2]. Many psychological processes have been shown to exacerbate outgroup derogation, including prejudicial attitudes and stereotyping [3,4]. Over the last 25 years, an increasing body of research has investigated the extent to which a psychological process of dehumanization increases the risk of intergroup harm [5–20]. According to the dehumanization hypothesis, when members of an outgroup are perceived as less human than ingroup members, they are at greater risk of harm [5,8,21–24]. Subtle forms of dehumanization are thought to be pervasive in contemporary society. For example, the dehumanization of national groups [25,26], religious groups [27,28], individuals on a low income [29] and refugees [30,31] has been reported in the literature.

Within social psychology, several different characterizations of dehumanization have been proposed. Infrahumanization theory [32,33] posits that a subtle form of dehumanization occurs where outgroup members are viewed as experiencing uniquely human emotions such as pride and melancholy to a lesser extent than ingroup members. The mental state account maintains that outgroup members are dehumanized to the extent that they are denied mental states [34–36].

The dual model of dehumanization is of particular interest to the current research [37]. According to the dual model, individuals and groups are dehumanized to the extent that they are denied uniquely human character traits. The dual model distinguishes between two forms of dehumanization [38]. When outgroup members are *animalistically dehumanized*, or perceived as similar to animals, they are thought to possess traits such as civility, refinement, rationality, moral sensibility and maturity to a lesser extent than the ingroup. When outgroup members are *mechanistically dehumanized*, or perceived as similar to robots, they are thought to possess traits such as emotional responsiveness, interpersonal warmth, depth, cognitive openness and agency to a lesser extent than the ingroup.

According to the dual model, the more an individual or group is either animalistically or mechanistically dehumanized, the greater their risk of being harmed [23,37]. Haslam & Loughnan [23] argue that *‘dehumanization is important as a psychological phenomenon because it can be so common and yet so dire in its consequences’* (p. 401). Haslam [39] further notes that *‘Many studies have examined how dehumanizing perceptions enable harm or provide support for it. Some of this work points to direct links between tendencies to dehumanize others and*… *aggressive behaviour’* (p. 139). Empirical research has suggested that trait-based dehumanization facilitates social exclusion [40] and reduces prosocial behaviour [41].

Bastian *et al*. [42] conducted influential empirical studies testing the hypothesized association between the denial of human character traits and the endorsement of harsh punishment [43–48]. The researchers measured how trait-based dehumanization influenced participants’ punishment of criminals. Participants were asked to rate their agreement with four items assessing animalistic dehumanization of criminals: *‘I felt like the person in the story was refined and cultured’* [*reversed*], *‘I felt like the person in the story was rational and logical, like they were intelligent’* [*reversed*], *‘I felt like the person in the story lacked self-restraint, like an animal’ and ‘I felt like the person in the story was unsophisticated’*. Participants were also asked to rate their agreement with four items assessing mechanistic dehumanization of criminals: *‘I felt like the person in the story was open minded, like they could think clearly about things’* [*reversed*], *‘I felt like the person in the story was emotional, like they were responsive and warm’* [*reversed*], *‘I felt like the person in the story was superficial like they had no depth*’ and *‘I felt like the person in the story was mechanical and cold, like a robot’*. Bastian *et al*. [42] reported that both forms of dehumanization predicted endorsement of harsh punishment for the criminals portrayed in their stimuli, concluding that their participants viewed criminals as *‘subhuman and beastly’* (p. 9).

Recently, however, the explanatory value of the dual model has been called into question [5,6,49]. According to these critiques, evidence for trait-based dehumanization is often confounded with social desirability. In Bastian *et al*.’s work [42], evidence that criminals were animalistically dehumanized was drawn from the observation that participants judged them to be unsophisticated, lacking self-restraint, unrefined, uncultured, irrational and unintelligent. Evidence that criminals were mechanistically dehumanized came from the observation that participants viewed them as superficial, cold and lacking in warmth and responsiveness. These results may reflect dehumanization because the traits criminals were found to lack are those perceived as uniquely or essentially human [37,38]. However, as the traits deemed uniquely human were all socially desirable, evidence for trait-based dehumanization cannot be separated from evidence of negative evaluation more generally. An alternative explanation for the findings of Bastian *et al*. [42] is that participants endorse harsh punishment against criminals to the extent they perceive criminals to possess undesirable or antisocial characteristics.

Bastian *et al*. [42] seek to account for this possibility by statistically controlling for participants’ moral outrage at the targets’ behaviour in their analysis. They report that the relationship between trait-based dehumanization and punishment remains even when moral outrage is controlled for. While this is interesting and suggestive of the independent effects of dehumanization, it cannot fully address the conceptual weaknesses in how dehumanization was operationalized. A more convincing way to de-confound evidence for trait-based dehumanization from evidence of negative evaluation is to ask participants to rate the target group on traits that are uniquely human but vary from socially desirable to undesirable [5,6]. Previous research conducted by Enock *et al*. [49] has established that undesirable character traits such as *jealous*, *spiteful* and *bitter* are considered unique to humans and socially undesirable. Across three intergroup contexts, the researchers found that participants attributed socially desirable human traits more strongly to the ingroup and socially undesirable traits more strongly to the outgroup; see also [50–53]. Enock *et al*. [49] concluded that intergroup preference may better explain apparent evidence for trait-based dehumanization. However, it is not yet clear how the attribution of uniquely human character traits relates to harm. Addressing this question is crucial to understanding the extent to which the dual model of dehumanization can help explain real-world discrimination and negativity.

We revisit the hypothesized causal relationship between trait-based dehumanization and harm in the context of endorsing harsh punishment for criminals. In studies 1A and 1B, we seek to conceptually replicate the key findings of Bastian *et al*. [42], suggesting that the extent to which participants animalistically (study 1A) and mechanistically (study 1B) dehumanize criminals predicts the severity of the punishment participants endorse for them. In studies 2A and 2B, we adopt a similar design but incorporate socially undesirable traits into our stimulus set. This addition to the design allows us to investigate whether trait-based dehumanization, undesirable trait attribution, or both predict the severity of punishment. Following Bastian *et al*., and to understand the generalizability of our findings, we investigate these questions in relation to two different types of crime (violent crime and theft). In studies 3A and 3B, we seek to investigate a similar question using an experimental design and focusing on parole decisions rather than sentencing. We present participants with vignettes in which criminals are described using character traits that differ in how socially desirable they are and whether they are unique to humans. We then measure how these varying descriptions influence participants’ parole decisions. This design allows us to directly measure whether there is a causal relationship between trait-based dehumanization and punishment, independent of an effect of ingroup preference.

### 1.1. Methods

All studies received ethical approval from the Psychology Departmental Ethics Committee at the University of York (approval no. 926). All data collection occurred online, and the studies were created and administered using Qualtrics (https://www.qualtrics.com). Participants were recruited through the online platform Prolific (https://www.prolific.co), with an independent sample recruited for each study. Informed consent was obtained at the start of each session according to approved ethical guidelines. Inclusion criteria for each study included adult participants fluent in English who had never been to prison for committing a crime and had a Prolific approval rating of at least 90% (95% for studies 3A and 3B). Increases in Prolific’s recommended rate of compensation for participation during data collection meant the reward ranged from approximately £7 per hour in studies 1A and 1B to approximately £8 in the other four studies. Assumption testing and analyses were conducted using SPSS and RStudio. All studies were pre-registered on AsPredicted.com before commencing data collection. Links to pre-registration documents, data files [54], a fully computationally reproducible version of the manuscript, and electronic supplementary materials, including the stimuli used for each study, can be found at: http://doi.org/10.17605/OSF.IO/D4CVP.

## 2. Study 1A

Bastian *et al*. [42] presented evidence that the more participants dehumanize violent criminals, the harsher the punishment participants endorse for them. We sought to test whether we could conceptually replicate this relationship between trait-based dehumanization and punishment using terms similar to those used by Bastian *et al*. [42]. In study 1A, participants read a series of scenarios describing fictitious criminals and their violent crimes. Following this, participants rated the extent to which the criminals possessed four character traits that distinguish humans from non-human animals (*refined*, *rational and logical*, *has a sense of morality* and *civilized*), which we refer to as uniquely human traits. Participants also rated the extent to which criminals possessed four character traits that distinguish humans from machines (*open-minded*, *emotionally responsive*, *has a depth of character* and *interpersonally warm*), which we refer to as human nature traits. In investigating traits that distinguish humans from animals and machines, we are respecting the distinction between animalistic dehumanization and mechanistic dehumanization which is basic to the dual model [37, p. 256]. Participants also responded to an item measuring how harsh they thought the violent criminals’ punishment should be. Following Bastian *et al*. [42], we predicted that the fewer participants attributed these uniquely human traits to criminals, the harsher the punishment they would recommend for criminals.

### 2.1. Methods

#### Participants

A power analysis using G*Power indicated that a sample size of 89 would allow us to detect a medium effect size (*f* ^2^ = 0.15) with an alpha of 0.05 and a power of 0.95. A final sample of 100 participants was collected, with 54 identifying as female, 44 as male and two as non-binary. Ages ranged from 18 to 63 (*M* = 26.5, s.d. = 8.94). In accordance with our pre-registered exclusion criteria, data submitted by six individuals who failed one or both attention checks (i.e. gave a response more than 20 points away from the instructed end of the scale) were omitted and replaced. Participation took an average of approximately 8 min.

#### Materials

#### Vignettes

All participants responded to the same five vignettes detailing different scenarios involving violent crimes. An effort was made to ensure that all five vignettes were similar in length, degree of detail and severity of crimes depicted. In each vignette, the target criminal’s age and ethnicity were not indicated, and the scenarios depicted were all set in unspecified locations. Each target’s name and pronouns were gender-neutral, though we cannot rule out assumptions made by participants about gender. All vignettes are included in the electronic supplementary materials. For example, *Charlie was arrested after a fight broke out in a pub soon after opening time, apparently triggered by a minor disagreement. Charlie smashed a pint glass and used it to stab another customer. Two additional customers received cuts as they tried to hold Charlie back until the Police arrived.*

#### Trait attribution

After reading each vignette, participants responded to items designed to measure trait-based dehumanization, broadly following the procedure of Bastian *et al*. [42]. Participants indicated the extent to which they attributed four uniquely human traits (refined, rational and logical, has a sense of morality and civilized) and four human nature traits (open-minded, emotionally responsive, has a depth of character, and interpersonally warm) to the criminals depicted. Participants indicated their agreement with each item (e.g. *I think* [e.g. *Charlie*] *is refined*) using an unmarked sliding scale from 0 (*Strongly Disagree*) to 100 (*Strongly Agree*), with the sliders initially fixed at the midpoint. According to the dual model, lower scores indicated greater dehumanization of violent criminals. An attention check appeared halfway through the dehumanization items for two criminals (*Please move the slider all the way to Strongly Agree*/*Disagree*).

#### Harshness of punishment endorsed

Using an unmarked sliding scale that ranged from 0 (*Not at all harsh*) to 100 (*Very harsh)*, participants were asked to respond to the question *How harsh do you think the punishment for* [e.g. *Charlie*] *should be*?

#### Design

Following Bastian *et al*. [42], we used a within-subjects, correlational design. All participants read the same five vignettes presented in random order and responded to the same trait attribution and punishment items. Participants’ scores for the trait attribution items and the endorsed harshness of punishment item were then averaged across scenarios. The presentation of the items in the trait attribution task was also randomized.

#### Procedure

Participants were informed that the study would examine how social attributions influence our behavioural intentions towards criminals. After providing informed consent, participants answered a few demographic questions and confirmed that they had never been to prison for committing a crime. The first of five vignettes then followed. After reading the vignette, participants were asked to respond to the trait attribution items, followed by the single item asking them to indicate how harshly they thought the criminal should be punished. Participants repeated the above steps for each of the remaining four vignettes. To ensure participants read the stimuli carefully, each vignette remained on the screen for at least 15 s.

### 2.2. Results

#### Model 1: Animalistic dehumanization and punishment

**Figure 1.**
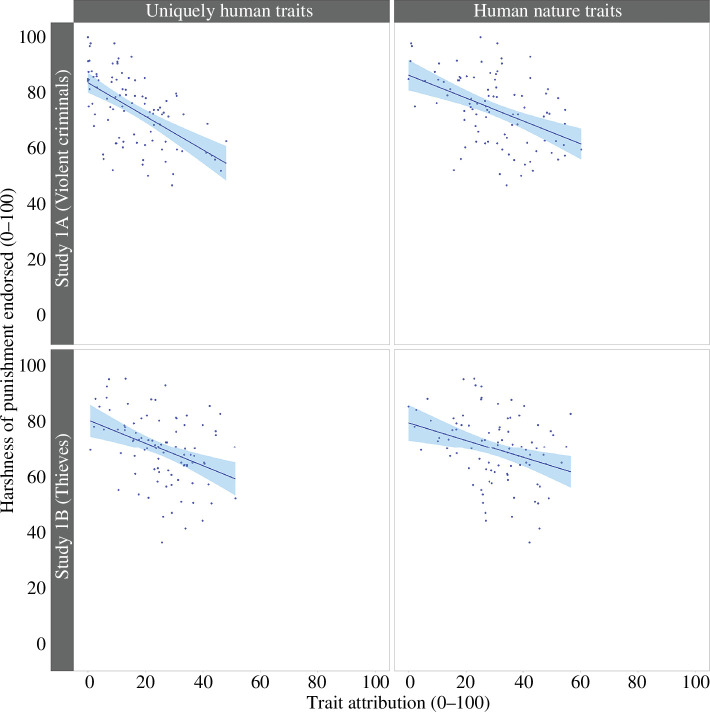
Results of studies 1A and 1B: seemingly in line with Bastian *et al*. [42], greater animalistic (left) and mechanistic (right) dehumanization of violent criminals (study 1A, top) and thieves (study 1B, bottom) was associated with harsher punishment.

In line with our pre-registered criteria, this analysis omitted two highly influential cases (remaining sample *n* = 98). We first calculated the average attribution score for uniquely human traits and punishment for each participant in the sample. We then conducted a simple linear regression to understand whether the extent to which participants attributed uniquely human traits to criminals predicted the harshness of punishment participants endorsed for them. A significant negative relationship was found, *b* = −0.56 [−0.75, −0.37], *t* = −5.93, *p* < 0.001; see figure 1. Thus, the more violent criminals were animalistically dehumanized (by being denied uniquely human traits), the harsher the punishment participants endorsed. The model explained approximately 27% of the variance in the harshness of punishment scores, *R^2^* = 0.27, *F*(1,96) = 35.14.

#### Model 2: Mechanistic dehumanization and punishment

In line with our pre-registered criteria, seven highly influential cases were omitted from the analysis (remaining sample *n* = 93). After calculating the average attribution score for human nature traits and punishment for each participant, we conducted a simple linear regression to test whether attribution of human nature traits predicted the harshness of punishment endorsed for violent criminals. A significant negative relationship was found, *b* = –0.41 [–0.57, –0.24], *t* = −4.928, *p* < 0.001; see figure 1.

This relationship shows that greater mechanistic dehumanization (operationalized as the denial of human nature traits) was associated with the endorsement of harsher punishment. The model explains 21% of the variance in the harshness of punishment scores, *R^2^* = 0.21, *F*(1,91) = 24.29.

## 3. Study 1B

Study 1B investigates whether the relationship found in study 1A replicates when participants are asked to judge a different type of criminal activity. In study 1B, we examined whether animalistic and mechanistic forms of dehumanization, as operationalized by Bastian *et al*. [42], are associated with the harshness of punishment endorsed for individuals who commit theft. The design, materials and analysis plan were similar to that used in study 1A, except that the scenarios involved theft rather than violent crime. In investigating a different type of crime, we follow the example set by Bastian *et al*. and seek to understand the generalizability of our results.

### 3.1. Method

#### Participants

Based on the same power analysis used in study 1A, a sample of 100 participants was collected, with 55 identifying as male and 45 as female. Ages ranged from 18 to 57 (*M* = 25.5, s.d. = 8.10). The attention checks in study 1A were also used in study 1B. Ten participants failed one or both attention checks, and their data were omitted and replaced as per our pre-registration. Participation took an average of 9 min.

#### Materials

The measures of dehumanization and punishment were identical to those used in study 1A.

#### Vignettes

All participants responded to the same five vignettes, each detailing a crime involving theft (see electronic supplementary materials). As in study 1A, an effort was made to ensure the vignettes were similar in structure and amount of detail. Once again, all of the perpetrators had gender-neutral names. An example of one of the theft vignettes is as follows: *Until their recent arrest, Charlie had worked as a till operator at a local charity shop supporting individuals experiencing homelessness. Charlie had been stealing cash amounts varying from £5 to £50 from the tills almost daily over a five-year period. Police revealed that Charlie had stolen several thousand pounds from the charity shop while working there*.

### 3.2. Results

#### Model 1: Animalistic dehumanization and punishment

Seven highly influential cases were omitted from the analysis (remaining sample *n* = 93). We calculated the average attribution scores for uniquely human trait attribution and punishment for each participant and then conducted a simple linear regression to measure whether trait attribution predicted the harshness of punishment endorsed for thieves. As shown in figure 1, a significant negative relationship was found, *b* = –0.42 [–0.64, –0.21], *t* = −3.97, *p* < 0.001. Thus, greater animalistic dehumanization of thieves was associated with the endorsement of harsher punishment for them. The model explains approximately 15% of the variance in the harshness of punishment scores, *R^2^* = 0.15, *F*(1,91) = 15.75.

#### Model 2: Mechanistic dehumanization and punishment

Eight highly influential cases were omitted from the analysis (remaining sample *n* = 92). After calculating the average score of human trait attribution and punishment, we conducted a simple linear regression to test whether or not human trait attribution predicted the harshness of punishment endorsed for thieves. A significant negative relationship was found, *b* = −0.27 [−0.47, −0.08], *t* = −2.77, *p* = 0.007. These data show that greater mechanistic dehumanization is associated with the endorsement of harsher punishment for thieves (see figure 1). The model explains approximately 8% of the variance in the harshness of punishment scores, *R^2^* = 0.08, *F*(1,90) = 7.65.

## 4. Study 2A

Study 2A investigated whether apparent evidence for a relationship between trait-based dehumanization and endorsement of harsh punishment for violent criminals remains when controlling for the desirability of the traits. We tested this by introducing character traits perceived as uniquely human yet socially undesirable into the stimulus set [5,6,49]. The dual model predicts that to the extent criminals are denied uniquely human character traits, they will be subjected to harsher punishment. We predict that trait desirability will moderate the relationship between human trait attribution and punishment. More specifically, we predict that the extent to which violent criminals are denied socially desirable character traits and attributed socially undesirable character traits will predict harsh punishment.

### 4.1. Method

#### Participants

A power analysis using G*Power indicated that a sample size of 119 would allow us to detect a medium effect size (*f^2^* = 0.15), with three predictors (trait attribution; trait desirability; attribution∗desirability), an alpha of 0.05 and power of 0.95. To counterbalance the sample equally and allow for the exclusion of outliers, a sample of 130 was collected. Within the sample, 66 identified as female, 62 as male, and two as non-binary. Ages ranged from 18 to 55 (*M* = 28.5, s.d. = 9.15). Similar to studies 1A and 1B, two attention checks were included in this study. Per our pre-registered plan, 16 participants failed one or both attention checks; thus, their data were omitted and replaced. Participation took an average of nearly 8 min.

#### Design

This study used a mixed design. All participants responded to items designed to measure animalistic dehumanization and mechanistic dehumanization. The desirability of the traits rated by participants was manipulated between subjects: half of the participants rated criminals on the extent to which they possessed socially desirable traits, and half rated criminals on the extent to which they possessed undesirable traits. All participants responded to a single item measuring the harshness of punishment endorsed.

#### Materials

#### Vignettes

All participants read the same vignettes describing violent crimes as in study 1A.

#### Trait attribution

After reading each vignette, participants responded to an eight-item scale measuring animalistic dehumanization (four items) and mechanistic dehumanization (four items) of the criminal portrayed. Participants made trait attributions by indicating their agreement with each item using an unmarked sliding scale ranging from 0 (*Strongly Disagree*) to 100 (*Strongly Agree*), all of which were initially positioned at the scale’s midpoint. Depending on the condition, the eight trait items were either socially desirable (uniquely human: *cultured, civilized, sophisticated, moral*; human nature: *generous, open-minded, warm, kind*) or socially undesirable (uniquely human: *corrupt, controlling, arrogant, bitter*; human nature: *jealous selfish, spiteful, cruel*). The lower the score, the more participants dehumanize the criminal target by denying them human traits.

#### Harshness of punishment endorsed

The same single-item scale for measuring the harshness of punishment endorsed in studies 1A and 1B was employed in study 2A.

#### Procedure

The procedure in study 2A mirrored that of studies 1A and 1B.

### 4.2 Results

#### Model 1: animalistic dehumanization and punishment

Eight highly influential cases were omitted from the analysis (remaining sample *n* = 122). The regression model tested for a relationship between participants’ average scores for uniquely human trait attribution and harshness of punishment endorsed with trait desirability included as a moderator (desirable = 0, undesirable = 1).

The moderated regression showed no significant effect of uniquely human trait attribution on punishment, *b* = −0.07 [−0.22, 0.09], *t* = −0.83, *p* = 0.408. Thus, when undesirable uniquely human traits were included in the measure of animalistic dehumanization, the previously reported relationship between animalistic dehumanization and the endorsement of harsher punishment [42] was no longer significant.

**Figure 2.**
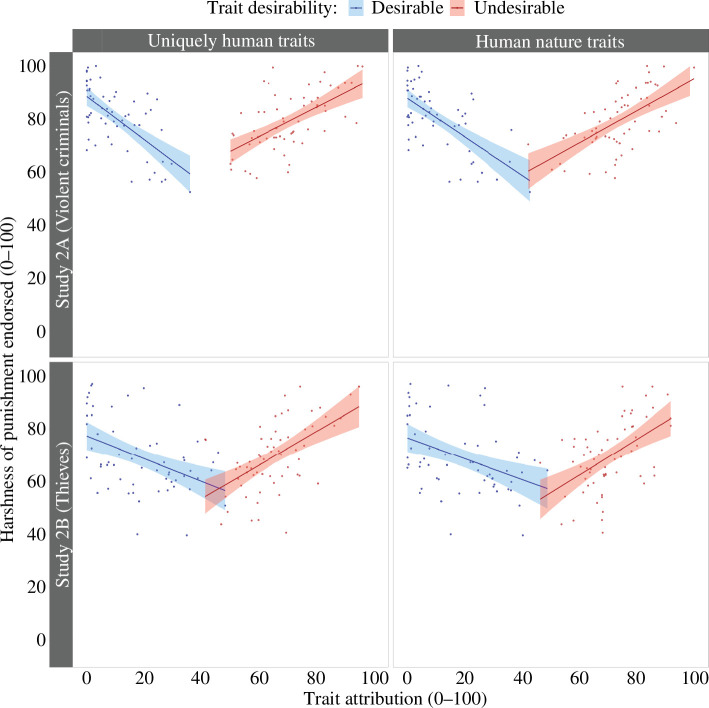
Results of studies 2A and 2B: the relationship between trait attribution and punishment for violent criminals (study 2A, top) and thieves (study 2B, bottom) depends on the social desirability of the traits.

The interaction between uniquely human trait attribution and trait desirability was significant, *b* = 1.31 [1.00, 1.63], *t* = 8.31, *p* < 0.001. In line with our prediction, simple slopes showed that the more socially desirable human traits participants attributed to criminals, the less harshly participants thought they should be punished, *b* = −0.58 [0.15, 0.52], *t* = −5.90, *p* < 0.001. The more undesirable traits participants attributed to criminals, the more harshly participants thought they should be punished, *b* = −0.73 [−0.98,−0.49], *t* = −5.92, *p* < 0.001 (see figure 2). The model explained approximately 38% of the variance in the harshness of punishment endorsed, *R^2^* = 0.375, *F*(3,118) = 23.61.

#### Model 2: mechanistic dehumanization and punishment

Eight highly influential cases were omitted from the analysis (remaining sample *n* = 122). A moderated regression analysis tested for a relationship between the average scores of human trait attribution and harshness of punishment endorsed to violent criminals and whether this interacted with trait desirability.

The moderated regression showed no significant effects of human nature trait attribution on punishment, *b* = −0.04 [−0.19, 0.12], *t* = −0.44, *p* = 0.658. The effect reported by Bastian *et al*. [42], whereby mechanistic dehumanization predicted harsher punishment endorsement, which we replicated in studies 1A and 1B, did not appear when undesirable human nature traits were included in our measures.

The interaction between uniquely human trait attribution and trait desirability was significant, *b* = 1.37 [1.06, 1.68], *t* = 8.65, *p* < 0.001. Simple slopes indicated that the more participants attributed socially desirable traits to criminals, the less harshly they thought those criminals should be punished, *b* = −0.73 [−0.96,−0.51], *t* = −6.46, *p* < 0.001. As shown in figure 2, the more participants attributed socially undesirable traits to criminals, the more harshly they thought those criminals should be punished, *b* = 0.64 [0.42, 0.86], *t* = 5.77, *p* < 0.001. The model explained 39% of the variance in the harshness of punishment scores, *R^2^* = 0.389, *F*(3,118) = 25.05.

### 5. Study 2B

Study 2B sought to replicate the results of study 2A but with thieves as the target group rather than violent criminals. We examined whether the apparent relationship between trait-based dehumanization and the endorsement of harsh punishment for thieves is better explained by the desirability of the traits incorporated into the stimulus set. We investigate this question using a very similar design and procedure to study 2A, with the exception that the vignettes are those used in study 1B detailing crimes involving theft. As in study 2A, we hypothesize that trait desirability will moderate the relationship between human trait attribution and punishment. More specifically, we predict the extent to which criminals are denied socially desirable character traits and attributed socially undesirable character traits will predict endorsement of harsher punishment.

### 5.1. Method

#### Participants

The power analysis described in study 2A informed the sample size for study 2B. A separate sample of 130 participants was collected, of whom 74 identified as male, 53 as female and three as non-binary. Ages ranged from 18 to 59 (*M* = 26.6, s.d. = 7.48). Data submitted by 20 participants who did not pass one or both checks were omitted and replaced. Participation took an average of 9.5 min.

#### Design

This study used a mixed-methods design, matching that of study 2A. The same attention checks used in studies 1A, 1B and 2A were used in study 2B.

### Materials

#### Vignettes

All participants responded to the same five vignettes used in study 1B detailing scenarios involving criminals committing theft.

#### Trait attribution

The same scales for measuring animalistic dehumanization, mechanistic dehumanization and punishment used in study 2A were used in study 2B.

#### Procedure

The procedure in study 2B was identical to that of study 2A, except for the vignettes describing crimes involving theft rather than violence.

### 5.2. Results

#### Model 1: animalistic dehumanization and punishment

Eight highly influential cases were omitted from the analysis (remaining sample *n* = 122). A moderated regression tested for a relationship between average scores of uniquely human traits and harshness of punishment endorsed and whether this interacted with trait desirability. The moderated regression showed no significant effects of uniquely human trait attribution on punishment *b* = 0.13 [−0.05, 0.30], *t* = 1.44, *p* = 0.152. Replicating the results of study 2A, when socially undesirable traits were incorporated into the stimulus set, there was no longer any relationship between trait-based dehumanization and punishment.

The interaction between uniquely human trait attribution and trait desirability was significant, *b* = 1.08 [0.74, 1.42], *t* = 6.22, *p* < 0.001. As illustrated in figure 2, the more participants attributed socially desirable traits to criminals, the less harshly participants felt they should be punished, *b* = −0.43 [−0.65,−0.21], *t* = −3.88, *p* < 0.001. The more participants attributed undesirable traits to criminals, the more harshly participants felt they should be punished, *b* = 0.65 [0.38, 0.91], *t* = 4.85, *p* < 0.001. The model explained about 25% of the variance in endorsed harshness of punishment scores, *R^2^* = 0.247, *F*(3,118) = 12.89.

#### Model 2: mechanistic dehumanization and punishment

The analysis omitted six highly influential cases (remaining sample *n* = 124). A moderated regression tested for a relationship between human trait attribution and punishment and whether this interacted with trait desirability. As in study 2A, and contradicting the findings of Bastian *et al*. [42], the moderated regression showed no significant relationship between human trait attribution and punishment, *b* = 0.15 [−0.04, 0.34], *t* = 1.57, *p* = 0.119.

However, the interaction between human nature trait attribution and trait desirability was significant, *b* = 0.95 [0.57, 1.33], *t* = 5.00, *p* < 0.001. As can be seen in figure 2, simple slopes showed that the more participants attributed socially desirable human traits to criminals, the less harshly participants thought they should be punished, *b* = −0.33 [−0.55,−0.11], *t* = −2.99, *p* = 0.003. The more participants attributed socially undesirable human traits to criminals, the more harshly participants thought they should be punished, *b* = 0.62 [0.31, 0.92] *t* = 4.01, *p* < 0.001. The model explained approximately 17% of the variance in the harshness of punishment scores, *R^2^* = 0.174, *F*(3,120) = 8.44. These data suggest that the apparent relationships between animalistic and mechanistic dehumanization and punishment reported in previous research [42] are better explained by the social desirability of the traits.

## 6. Study 3A

In study 3A, we used an experimental design to examine further the hypothesized causal relationship between trait-based dehumanization and punishment when controlling for the social desirability of human traits incorporated into the stimuli. We described criminals with traits that varied in desirability and perceived humanness, creating a 2 × 2 design. We then measured participants’ willingness to endorse parole for each criminal described. The dual model predicts that criminals who are described in uniquely human terms will be more likely to be granted parole. We predicted that criminals described in socially desirable terms would be more likely to be granted parole. In principle, this design allows us to detect independent effects of dehumanization and trait sociability or an interaction between the two. In study 3A, we specifically measure the extent to which animalistic dehumanization is causally related to parole decisions. Thus, our measures included uniquely human traits and those shared with other animals. We predict that participants will be more likely to endorse parole for criminals described with socially desirable traits, regardless of whether or not those traits are uniquely human or shared with other animals.

### 6.1. Method

#### Participants

A power analysis using G*Power, with effect size specification as in SPSS, indicated that a sample size of 135 would allow us to detect a medium effect size (*η*_p_^2^ *=* 0.09) with a 2 × 2 factorial, repeated measures design, an alpha of 0.05, and a power of 0.95. To counterbalance the sample equally, a sample of 136 participants was collected, of whom 78 identified as female, 55 as male, two as non-binary, and one who preferred not to indicate their gender identity. Ages ranged from 18 to 63 (*M* = 24.9, s.d. = 6.97). All participants were adults fluent in English who had never been to prison for committing a crime. Owing to a noticeable increase in failed attention checks during pilot data collection, the minimal approval rating on Prolific was raised from 90 to 95%. Despite this, data from 46 participants were omitted and replaced owing to failed attention checks.^1^ Three participants were mistakenly recruited after the intended sample size had been met, and thus, their data were excluded from analyses. Including data submitted by excess participants in analyses yielded the same results as those reported. Participation took an average of 7 min.

### Materials

#### Vignettes

All participants responded to the same four vignettes, each detailing a different scenario in which a criminal’s eligibility for parole was assessed. Efforts were made to ensure that all four vignettes were similar in length, degree of detail and contextual aspects, such as how long the criminal had spent in prison and who was described as attributing the traits to the criminal. In each vignette, the criminal’s name and pronouns were gender-neutral, their age and ethnicity were not indicated, and their crime and sentence were not specified. The four vignettes for study 3A can be seen in the electronic supplementary materials. In the uniquely human socially desirable condition, the criminal was described as *cultured*, *civilized*, *sophisticated* and *moral*, while in the uniquely human condition socially undesirable, the criminal was described as *corrupt*, *controlling*, *arrogant* and *bitter*. In the animalistic desirable condition, the criminal was described as *energetic*, *trusting*, *genuine* and *having curiosity*, while in the animalistic undesirable condition, the criminal was described as *uncultured*, *unrefined*, *unsophisticated* and *stupid*.

The following is an example of a vignette describing a criminal with uniquely human, socially desirable traits: *Alex, known by locals in their hometown as having always been sophisticated, has recently begun their first parole hearing at the local courthouse. Having been tried and convicted 36 months ago, a report by one of the prison’s counsellors notes that other prisoners often refer to Alex as being civilized and moral in character. Alex was also described by the counsellor as exhibiting a cultured demeanour since their arrival*.

#### Agreement with parole

The dependent variable, agreement with granting parole, was measured using the following single-item measure: *I think (Alex/Sam/Robin/Jamie) should be granted parole*. This measure appeared after each vignette, and participants indicated their agreement using an unmarked sliding scale ranging from *Strongly Disagree* (0) to *Strongly Agree* (100). The slider’s starting point was always centred at 50.

#### Attention check

An additional paragraph describing a criminal named Charlie was included, largely similar to the other four paragraphs. However, in the middle of the paragraph, the following sentence was included: *This paragraph is an attention check: please move the slider all the way to Strongly Disagree on the left-hand side*. Data submitted by any participants who did not respond within 20 points of the instructed end of the 100-point scale were omitted and replaced.

#### Design

This study adopted a 2 (trait humanness: uniquely human, shared) × 2 (trait desirability: desirable, undesirable) within-subjects factorial design. Counterbalancing ensured that each vignette was associated with each trait category an equal number of times across the participant sample, resulting in four trait-type orders. The trait words were randomly allocated to the position in which they appeared in each vignette using a random order function in Excel. Mirror versions of the trait orders were then created. These two trait-order conditions were also counterbalanced between participants, which was done to control for possible primacy and recency effects of the order in which traits appeared.

#### Procedure

After participants provided informed consent, they responded to the same demographic questions and inclusion checks as in the other studies. Participants were then shown the first of the four vignettes. After reading the vignette, participants were asked to respond to a single item measuring their agreement with granting parole to the criminal depicted. Participants then repeated the above steps for the remaining three vignettes. The order in which the vignettes were presented to participants was randomized. Each vignette appeared on the screen for at least 15 s to maximize the chance that participants read all the relevant information. Participants were debriefed and redirected to Prolific to collect their reward after completing the questionnaire.

### 6.2. Results

**Figure 3.**
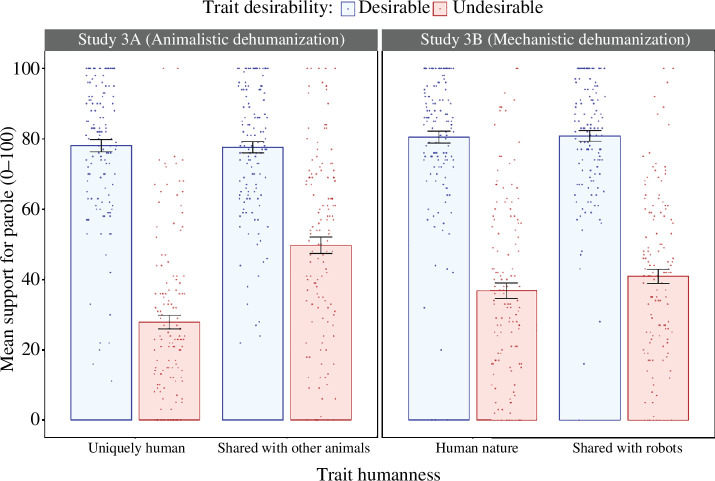
Results of studies 3A and 3B: criminals described with undesirable traits were less likely to be granted parole than criminals described with desirable traits, regardless of whether or not those traits were uniquely human. Error bars represent ±1 s.e.

A 2 × 2 within-subjects ANOVA was conducted to examine how variations in the desirability (desirable or undesirable) and humanness (uniquely human or shared with other animals) of the traits used to describe criminals influenced participants’ agreement with granting them parole. In line with our prediction, a significant main effect of trait desirability was found, *F*(1,135) = 369.43, *p* < 0.001, *η*_p_^2^ = 0.73. Criminals described with socially undesirable traits (*M* = 38.8, s.e. = 1.72) were less likely to be granted parole than were those described with desirable traits (*M* = 77.8, s.e. = 1.44); see figure 3. A main effect of trait humanness was also found, *F*(1,135) = 51.62, *p* < 0.001, *η*_p_^2^ *=* 0.28. Contrary to the predictions of the dual model, however, criminals who were described with uniquely human traits (*M* = 53.0, s.e. = 1.36) were less likely to be granted parole than those described with traits shared with other animals (*M* = 63.7, s.e. = 1.50).

A significant interaction between trait humanness and trait desirability was also found, *F*(1,135) = 54.67, *p* < 0.001, *η*_p_^2^ = 0.29. Paired samples *t-*tests with a Bonferroni corrected alpha level of 0.025 were conducted to examine interaction effects. Criminals described using undesirable uniquely human traits (*M* = 27.9, s.e. = 1.93) were less likely to be granted parole than were criminals described using desirable uniquely human traits (*M* = 78.0, s.e. = 1.70), *t*(135) = 20.75, *p* < 0.001, Cohen’s *d* = 1.78. Similarly, criminals described using undesirable traits shared with other species (*M* = 49.8, s.e. = 2.32) were less likely to be granted parole than were those described using desirable traits shared with other species (*M* = 77.6, s.e. = 1.6), *t*(135) = 10.57, *p* < 0.001, Cohen’s *d* = 0.91.

## 7. Study 3B

Study 3B had an extremely similar design and method to study 3A. We again employed an experimental manipulation in which we manipulated the perceived humanness and sociality of the traits with which criminals were described and measured how these descriptions influenced participants’ parole decisions. In study 3B, we specifically tested for an influence of mechanistic dehumanization by including human nature traits and traits shared with robots in our measures.

As in study 3A, we predicted that criminals described with undesirable traits would be less likely to be granted parole than those described using desirable traits.

### 7.1. Method

#### Participants

The power analysis described in study 3A also informed the sample size for study 3B. A new sample of 136 participants was collected, of whom 76 identified as female, 56 as male, two as non-binary, and two did not indicate their gender identity. Ages ranged from 18 to 57 (*M* = 26.4, s.d. = 8.29). The inclusion criteria were identical to those used in study 3A, including a minimum Prolific approval rating of 95%. Data from 35 participants were omitted and replaced owing to failed attention checks (see footnote 1). Five participants were mistakenly recruited after the intended sample size had been met, and thus, their data were excluded from analyses. Including data submitted by excess participants in analyses yielded the same results as those reported. Participation took an average of just under 7 min.

#### Materials

The agreement with granting parole scale and attention check were the same as those used in study 3A.

#### Vignettes

All participants responded to the same four vignettes used in study 3A but with different trait words. The desirable human words were *generous*, *open-minded*, *warm* and *kind*. The undesirable human words were *jealous*, *selfish*, *spiteful* and *stingy*. The desirable traits shared with robots were *helpful*, *disciplined*, *calm* and *efficient*. The undesirable traits shared with robots were *cold*, *inflexible*, *superficial* and *passive*. The following vignette is an example of the undesirable shared condition:

*Sam is currently applying for parole after being convicted of a crime just over three years ago. In assessing Sam’s suitability, the parole committee gathered reports from prison staff and other inmates. Guards patrolling the prison grounds noted Sam as being passive. Other prisoners mention Sam as exhibiting superficial behaviour with them for the most part. The prisoner who shares a cell with Sam has referred to them as the most inflexible cell-mate they have ever had. In last week’s parole hearing, Sam’s responses indicated a cold character*.

#### Design and procedure

The design and procedure were identical to that of study 3A.

### 7.2. Results

A 2 × 2 within-subjects ANOVA was conducted to examine how variations in trait humanness (human or shared with robots) and trait desirability (desirable or undesirable) influenced participants’ parole decisions. As illustrated in figure 3, a significant main effect of trait desirability was found, *F*(1,135) = 409.60, *p* < 0.001, *η*_p_^2^ = 0.75. Criminals described using undesirable traits (*M* = 38.9, s.e. = 1.82) were less likely to be granted parole than were criminals described using desirable traits (*M* = 80.6, s.e. = 1.44).

No significant main effect of trait humanness was found, *F*(1,135) = 2.782, *p* = 0.098, *η*_p_^2^ = 0.02. Participants were no more likely to grant parole to criminals who were described using human traits (*M* = 58.7, s.e. = 1.53) than those described using traits shared with robots (*M* = 60.8, s.e. = 1.33). Unlike in study 3A, no interaction effect between trait humanness and trait desirability was found, *F*(1,135) = 2.49, *p* = 0.117, *η*_p_^2^ = 0.018.

## 8. General discussion

Across six highly powered and pre-registered studies, we examined the hypothesized causal relationship between trait-based dehumanization and harm. The dual model of dehumanization [37] posits that individuals and groups are sometimes subtly dehumanized by being denied human character traits. To the extent that groups are dehumanized in this way, they are thought to be vulnerable to harm [21,22]. The work of Bastian *et al.* [42] is often cited in support of this claim. Bastian *et al*. [42] reported that the fewer human traits participants attributed to criminals, the harsher the punishments participants endorsed for them.

We initially sought to replicate the dehumanization effect reported by Bastian *et al.* [42] in a conceptually similar design. In study 1A, we examined the relationship between animalistic and mechanistic dehumanization, as operationalized by Bastian *et al*. [42], and the harshness of punishment endorsed by participants. In both studies, we successfully replicated previous findings, demonstrating that our paradigm was sensitive to finding predictive relationships between trait-based dehumanization and harm should they occur.

In studies 2A and 2B, we investigated the extent to which the previously reported relationship between trait-based dehumanization and harm can be explained by the social desirability of the traits incorporated into the stimulus set. The dual model [37] has previously been critiqued for failing to clearly distinguish evidence for trait-based dehumanization from evidence of negative evaluation [5,6,12,49]. Bastian *et al*. [42] operationalized animalistic dehumanization as a reduction in the extent to which participants viewed criminals as possessing traits such as sophistication and refinement. They operationalized mechanistic dehumanization as a reduction in the extent to which participants viewed criminals as possessing traits like warmth and depth. As each of these human traits is socially desirable, it is unclear whether harm was predicted by dehumanization or negative evaluation. In order to tease apart the influence of dehumanization and negative evaluation in harm, we incorporated undesirable human traits into our stimulus set, for example, *bitter* and *spiteful*. If trait-based dehumanization explains harm, the previously reported relationship between dehumanization and punishment should remain even when undesirable human traits are incorporated into the stimulus set. If the previously reported relationship is better explained by negative evaluation, then trait desirability should moderate the relationship with punishment. In support of the latter claim, studies 2A and 2B showed that the more desirable human traits participants attributed to criminals, the less harshly participants thought they should be punished. The more undesirable human traits participants attributed to criminals, the more harshly participants thought they should be punished.

In the third pair of studies, we sought to further distinguish between these two competing hypotheses using an experimental manipulation. In studies 3A and 3B, we described criminals in traits that varied in perceived humanness and sociality and measured the influence of these varying descriptions on participants’ parole decisions. This experimental design allowed us to directly test the hypothesized causal relationship between trait-based dehumanization and punishment. Converging with the findings of study 2, we found that criminals described with undesirable traits were less likely to be granted parole than were criminals described with desirable traits, regardless of whether or not those traits were uniquely human. There was no evidence for the hypothesis that criminals described with uniquely human terms would be more likely to be granted parole.

These findings fit with broader critiques of social psychological models of dehumanization. Enock *et al*. [49] showed that what appears to be evidence for trait-based dehumanization of immigrants and political groups is better explained by negative evaluation. Similarly, Enock *et al*. [50] presented evidence that what appears to be emotion-based dehumanization of seven different outgroups is better explained by negative evaluation. In these studies, participants were more likely to attribute prosocial emotions to the ingroup regardless of whether they were uniquely human or not. Participants were more likely to attribute antisocial emotions to the outgroup, regardless of whether they were uniquely human or not. In further work, Enock & Over [51] presented evidence that the apparent relationship between emotion-based dehumanization and reductions in prosocial behaviour is better explained by negative evaluation.

Partially in response to these critiques, Kteily & Landry [56] presented a new social psychological model of dehumanization in which to dehumanize an individual or group is to perceive them as less than the ideal human. Under this characterization of dehumanization, to view a group as possessing negative attributes is to dehumanize them. However, to define dehumanization in such a broad way as any negative evaluation renders almost all social judgements dehumanizing [12]. It seems unlikely that we dehumanize our closest and most loved kin simply by perceiving their imperfections. It is crucial that future conceptual research on dehumanization more clearly delineates dehumanization from negative evaluation [5,6,12].

It is important to acknowledge that we considered only one target group in this study—criminals. We based this decision on the influence the findings of Bastian *et al*. [42] have had on the literature. However, there may be more evidence for the hypothesized causal relationship between trait-based dehumanization and harm in other intergroup contexts. In order to address this issue, future research should investigate the hypothesized relationship between trait-based dehumanization and harm in intergroup contexts that vary in the extent to which the outgroup is negatively evaluated. In addition to examining additional intergroup contexts, future research should also incorporate more trait terms into stimulus sets and present them in a range of different ways. Research on dehumanization has been critiqued for using relatively small stimulus sets [57]. Indeed, some studies have used a single trait term to assess dehumanization. For example, Leidner *et al*. [28] measured dehumanization by asking participants to rate the extent to which they agreed that members of the target outgroup experienced compassion. It will always remain possible that evidence for the causal relationship between dehumanization and harm could be found with a more sensitive design.

We are not trying to argue that trait-based dehumanization never occurs. Rather, our argument is considerably more modest. Taken in conjunction with other recent results, it is apparent that evidence for trait-based dehumanization has often been confounded with evidence for negative evaluation [5,6,12,49,50]. The results of the current study add to this growing body of critiques by showing that the findings of Bastian *et al*. [42], often cited as evidence for the claim that trait-based dehumanization leads to an increased risk of harm, are considerably less convincing than they first appear. It is imperative that future research tests whether there is evidence for trait-based dehumanization when trait desirability is controlled for, given the grave importance of understanding predictors of intergroup harm in the real world.

## Data Availability

All studies reported in this article were pre-registered and the data is available open access. Data files, pre-registration documents, a fully computationally reproducible version of the manuscript, and supplementary materials, including the stimuli used for each study, can be found at: [54].

## Footnotes

^1^
The high rate of failed attention checks was probably owing to a wider issue with Prolific at the time of data collection, a surge in new users after a social media post promoting the platform went viral [55].
